# Correction of Alkalemia in a Hemodialysis Patient: A Case Report

**DOI:** 10.7759/cureus.113556

**Published:** 2026-07-28

**Authors:** Ying H Luu, Hamid R Hajmomenian, Reza Khorsan

**Affiliations:** 1 Medicine/Nephrology, University of California Los Angeles David Geffen School of Medicine, Los Angeles, USA

**Keywords:** alkalemia, end-stage renal disease (esrd), hemodialysis, low-bicarbonate dialysate, respiratory alkalosis

## Abstract

Advanced chronic kidney disease, including end-stage renal disease (ESRD), typically leads to chronic progressive metabolic acidosis and acidemia due to reduced renal excretion of daily acid loads, particularly impaired ammonium excretion. Metabolic acidosis and acidemia typically develop as kidney function progressively declines. It is characterized by a low serum bicarbonate level and a high or normal anion gap. Alkalemia is relatively rare in ESRD but is a life-threatening condition. We present a case of a woman in her 70s with ESRD and severe alkalemia due to respiratory alkalosis, successfully treated with low-bicarbonate dialysate.

## Introduction

Normal human serum (blood) pH is tightly regulated between 7.35 and 7.45, with 7.40 being the ideal slightly alkaline set point for optimal physiological function. This narrow range is critical for enzyme activity, metabolic processes, and oxygen delivery [1,2].

Advanced chronic kidney disease (CKD), including end-stage renal disease (ESRD), typically leads to chronic progressive metabolic acidosis and acidemia due to reduced renal excretion of daily acid loads, such as ammonium excretion failure [3]. Metabolic acidosis and acidemia usually appear when the glomerular filtration rate (GFR) falls below 40 mL/min [3]. It is characterized by low serum bicarbonate (typically 12-22 mEq/L) and a high or normal anion gap [4]. Alkalemia (pH >7.45) in ESRD is relatively rare compared to acidemia but is a life-threatening condition [5].

Hemodialysis maintains serum pH, typically targeting 7.35-7.45, by using a bicarbonate-buffered dialysate (pH 6.9-7.6) to neutralize metabolic acidosis. The treatment adds bicarbonate to the blood, ensuring post-dialysis levels remain above 22 mmol/L to counteract acid accumulation [6]. Using different dialysate bicarbonate concentrations gives the clinician flexibility to balance the patient's acid-base disturbance.

We present a case of a female in her 70s with ESRD complicated by severe alkalemia due to respiratory alkalosis. While patients with ESRD typically suffer from acidosis, requiring higher-bicarbonate dialysate, her alkalemia was successfully corrected by modifying the dialysate bicarbonate concentration during hemodialysis. The dialysate bicarbonate concentration was reduced to 20 mmol/L to treat severe alkalemia.

## Case presentation

A woman in her 70s with ESRD on hemodialysis three times weekly, hypothyroidism, hypertension, respiratory failure status post tracheostomy, and ventilator dependence was admitted to the hospital for percutaneous endoscopic gastrostomy tube placement. Her procedure was complicated by dislodgment of the gastrostomy catheter, pneumoperitoneum, and sepsis. She was anuric. She did not have any history of vomiting, diarrhea, or other gastrointestinal (GI) loss. The patient was seen by surgery and infectious disease services.

The patient’s hospital course was complicated by bradycardia, severe hypotension, systolic blood pressure in the 40s mm Hg, and subsequent myoclonic jerking movements of face, extremities, and torso. The neurology service evaluated the patient. The patient underwent continuous electroencephalogram (EEG) and brain imaging studies. Clinical findings accompanied by EEG and brain imaging studies established the diagnosis of anoxic brain injury associated with generalized seizure. The patient was continued on hemodialysis treatment three times a week. The patient developed respiratory alkalosis and alkalemia following an anoxic brain injury due to hyperventilation and breathing above the ventilator-set respiratory rate.

The patient's chemistry panel is shown in Table 1.

**Table 1:** Metabolic panel over time H: high value, L: low value, GFR: glomerular filtration rate, AST: aspartate aminotransferase, SGOT: serum glutamic-oxaloacetic transaminase, ALT: alanine aminotransferase, SGPT: serum glutamic-pyruvic transaminase, CO₂: carbon dioxide

Metabolic panel	Day 0 7:37:00 AM	Day 1 6:00:00 AM	Day 2 6:36:00 AM	Day 3 6:52:00 AM	Day 4 11:40:00 AM	Day 5 7:34:00 AM	Day 6 5:58:00 AM	Day 7 5:33:00 AM	Day 8 5:32:00 AM	Day 9 4:19:00 AM	Day 10 4:50:00 AM	Day 11 5:33:00 AM	Day 12 5:50:00 AM	Day 13 5:11:00 AM	Day 14 6:28:00 AM	Day 15 6:26:00 AM	Day 16 7:33:00 AM	Day 17 8:24:00 AM
Sodium (mmol/L)	142	141	137	134 (L)	131 (L)	136	135	135	137	137	137	136	135	135	136	135	134 (L)	135
Potassium (mmol/L)	4.9	5.4 (H)	4.7	4.6	5	4.3	4.4	4.5	4.6	5	5.2	4.4	4.6	5.3	4.4	4.5	4.2	4.3
Choride (mmol/L)	100	97	98	104	100	103	100	100	99	108 (H)	105	107 (H)	105	103	107 (H)	106	109 (H)	107 (H)
Total CO2 (mmol/L)	26	27	27	20	18 (L)	19 (L)	18 (L)	21	22	18 (L)	17 (L)	15 (L)	14 (L)	14 (L)	16 (L)	15 (L)	16 (L)	15 (L)
Anion gap (mmol/L)	16	17	12	10	13	14	17	14	16	11	15	14	16	18	13	14	9	13
Urea nitrogen (mg/dL)	93 (H)	118 (H)	66 (H)	37 (H)	64 (H)	47 (H)	73 (H)	44 (H)	74 (H)	42 (H)	67 (H)	46 (H)	67 (H)	85 (H)	46 (H)	65 (H)	38 (H)	61 (H)
Creatinine (mg/dL)	4.00 (H)	4.72 (H)	2.87 (H)	2.09 (H)	3.08 (H)	2.24 (H)	3.08 (H)	2.03 (H)	3.00 (H)	1.96 (H)	2.88 (H)	2.20 (H)	3.02 (H)	3.75 (H)	2.35 (H)	3.24 (H)	2.14 (H)	2.99 (H)
Estimated GFR (mL/min/1.73m2)	11	9	17	25	16	23	16	26	16	27	17	23	16	12	21	15	24	16
Glucose (mg/dL)	201 (H)	239 (H)	186 (H)	192 (H)	174 (H)	174 (H)	162 (H)	234 (H)	162 (H)	191 (H)	143 (H)	95	98	61 (L)	149 (H)	148 (H)	160 (H)	161 (H)
Calcium (mg/dL)	10.7 (H)	10.6 (H)	10	10	10.2	9.9	10.4	9.8	10.2	10.1	10.3	10.2	10.3	10.2	9.6	10.1	10.1	10.2
Phosphorus (mg/dL)	4.2	4.9 (H)	4	4.3	5.8 (H)	4.8 (H)	6.2 (H)	4.2	5.7 (H)	4.3	5.6 (H)	4.8 (H)	5.7 (H)	6.6 (H)	4.3	6.2 (H)	3.9	5.1 (H)
Magnesium (mEq/L)	2.2 (H)	2.3 (H)	1.9	1.9	1.9	1.9	2.2 (H)	1.9	2.1 (H)	2.0 (H)	2.2 (H)	2.0 (H)	2.1 (H)	2.2 (H)	2.0 (H)	2.2 (H)	2.0 (H)	2.1 (H)
AST (SGOT) (U/L)	-	-	71 (H)	-	-	-	80 (H)	-	-	86 (H)	-	-	-	70 (H)	-	92 (H)	-	-
ALT (SGPT) (U/L)	-	-	19	-	-	-	24	-	-	34	-	-	-	23	-	34	-	-
Alkaline phosphatase (U/L)	-	-	356 (H)	-	-	-	385 (H)	-	-	380 (H)	-	-	-	324 (H)	-	378 (H)	-	-
Bilirubin, conj (mg/dL)	-	-	<0.2	-	-	-	<0.2	-	-	<0.2	-	-	-	<0.2	-	<0.2	-	-
Bilirubin, total (mg/dL)	-	-	0.4	-	-	-	0.3	-	-	0.3	-	-	-	0.3	-	0.3	-	-
Albumin (g/dL)	-	-	2.7 (L)	-	-	-	2.6 (L)	-	-	2.8 (L)	-	-	-	2.7 (L)	-	2.7 (L)	-	-
Total protein (g/dL)	-	-	8.2	-	-	-	8.2	-	-	8	-	-	-	7.6	-	7.8	-	-
Lactate (mg/dL)	-	-	-	-	-	-	-	-	-	16	-	-	-	-	-	-	-	-

The patient’s venous blood gas is shown in Table 2.

**Table 2:** Venous blood gas over time

Venous blood gas	Latest reference range and units	Day 1 6:07 AM	Day 1 10:24 AM	Day 2 7:02 AM	Day 2 10:55 AM	Day 3 6:55 AM	Day 4 11:48 AM	Day 5 7:53 AM	Day 6 6:10 AM	Day 7 5:48 AM	Day 8 5:37 AM	Day 9 4:39 AM	Day 11 5:43 AM	Day 12 6:14 AM	Day 13 5:26 AM	Day 14 6:37 AM
pH, venous	7.30-7.40	7.49 (H)	7.42 (H)	7.52 (H)	7.44 (H)	7.38	7.39	7.43 (H)	7.37	7.41 (H)	7.42 (H)	7.33	7.36	7.34	7.33	7.35
pCO2, venous	37-65 mm Hg	41	48	37	49	39	35 (L)	32 (L)	38	38	39	39	33 (L)	33 (L)	34 (L)	32 (L)
pO2, venous	No reference range mm Hg	67	50	75	38	59	65	60	61	48	60	59	86	77	64	66
Bicarbonate, venous	23.0-31.0 mmol/L	30.8	30.4	30.2	33.1 (H)	23.2	21.2 (L)	21.4 (L)	21.7 (L)	24	24.8	20.5 (L)	18.5 (L)	17.8 (L)	17.8 (L)	17.8 (L)
Base excess, venous	No reference range mmol/L	7	6	7	9	-2	-4	-3	-4	-1	0	-6	-7	-8	-8	-8
O2 sat/measured, venous	No reference range %	95.1	83.4	97	67.8	90.8	92.4	91.9	91.1	83.1	91.2	89.3	97.8	95.5	91.8	92.5

To correct alkalemia due to respiratory alkalosis, the patient underwent multiple hemodialysis sessions with low-bicarbonate dialysate (bicarbonate concentration of 20 mmol/L). The patient’s alkalemia resolved after using low-bicarbonate dialysate despite persistent respiratory alkalosis. Correction of the patient’s alkalemia based on venous bicarbonate can be seen in Figure 1.

**Figure 1:**
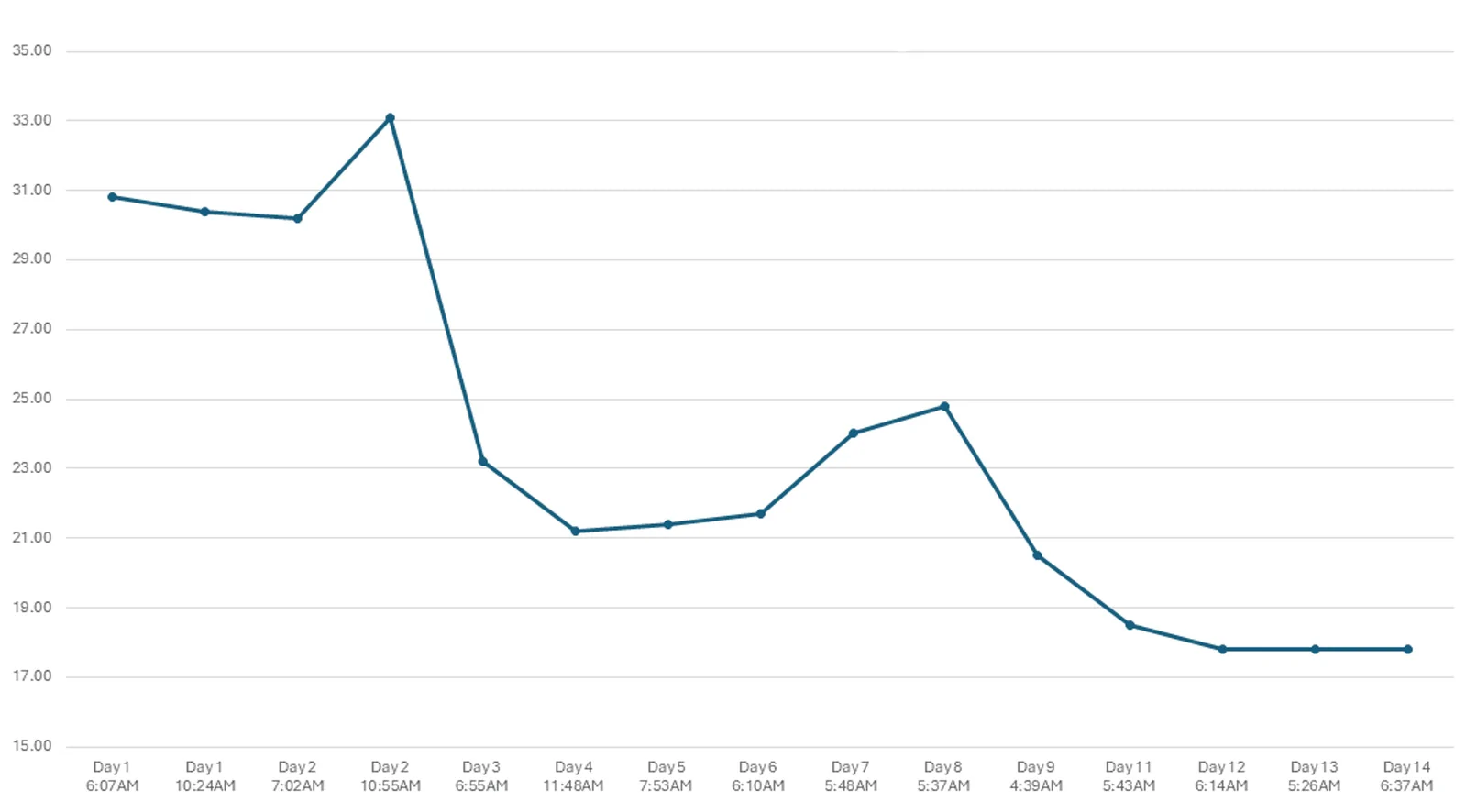
Change in venous bicarbonate over time The x-axis is time, and the y-axis is venous bicarbonate in mmol/L.

## Discussion

Acid-base disturbances are common in critically ill patients. Patients with CKD and ESRD predominantly develop chronic metabolic acidosis due to their reduced renal function. The severity of metabolic acidosis in patients with CKD correlates with the severity of their kidney dysfunction [7].

In normal kidney function, the kidneys maintain a normal plasma pH of 7.35-7.45 and a normal plasma bicarbonate level of 21-27 mmol/L by reabsorbing filtered bicarbonate as well as regenerating bicarbonate [8]. These two processes are impaired in patients with kidney disease. Metabolic acidosis is associated with several adverse consequences, including the worsening of renal function, bone disease, muscle wasting, and impaired growth [9].

In ESRD requiring renal replacement therapy (such as dialysis), thrice-weekly hemodialysis or daily peritoneal dialysis maintains normal plasma bicarbonate levels. In patients with reduced renal function who are not on renal replacement therapy, metabolic acidosis is treated by prescribing a base such as an alkalizing agent [6].

Alkalemia due to metabolic or respiratory causes is uncommon in patients with severely reduced renal function or patients with ESRD who are on renal replacement therapy [10]. While generally uncommon, alkalemia can occur. Alkalemia carries significant risks such as encephalopathy [9], decreased seizure threshold [11,12], cardiac arrhythmia [13], and electrolyte abnormalities such as hypocalcemia and hypokalemia [14].

Metabolic alkalosis (elevated pH and serum bicarbonate) is relatively rare in patients with ESRD, as they typically suffer from metabolic acidosis, but its prevalence is increasing. The primary causes for metabolic alkalosis include severe vomiting, constant nasogastric suctioning, use of inappropriately high-bicarbonate dialysate during dialysis, or intake of alkaline agents [15]. The patient in this case did not have any history of vomiting or any signs of GI loss.

Among acid-base disorders in patients with ESRD, respiratory alkalosis is also uncommon. However, respiratory imbalances are common, with studies showing an increased rate of respiratory alkalosis in hemodialysis patients along with various respiratory disturbances due to comorbidities, resulting in a mixed disorder with metabolic acidosis [16]. The patient in this case had severe alkalemia due to respiratory alkalosis.

Managing severe alkalemia (pH >7.55) due to respiratory alkalosis in a hemodialysis patient involves reducing hyperventilation, adjusting ventilator settings [17], if applicable, and modifying the dialysis prescription. Given the central hyperventilation due to recent anoxic brain damage in the reported case and overbreathing the ventilator-adjusted respiratory rate by the patient, sedation and/or adjustment of the ventilator settings were not appropriate in this case.

Hemodialysis using a low-bicarbonate dialysate (bicarbonate concentration of 20 mmol/L) was used in this case. The patient's severe alkalemia was corrected after the modified dialysis prescription. Correction of the patient's severe respiratory alkalosis and alkalemia by using low-bicarbonate dialysate during dialysis resulted in the anticipated low plasma bicarbonate.

## Conclusions

Alkalemia is not a common acid-base disturbance in patients with ESRD who are on dialysis. In patients with ESRD on hemodialysis, modifying the dialysate bicarbonate concentration provides flexibility to adjust the serum bicarbonate level, which in turn corrects serum pH. Clinicians must appreciate that correcting plasma pH is more important than correcting plasma bicarbonate in the setting of acid-base imbalance. Blood pH is the critical parameter for cellular function, enzyme activity, and oxygen delivery. While bicarbonate is crucial, the plasma pH directly indicates the severity of acidemia or alkalemia. While patients with ESRD typically suffer from acidosis, requiring higher-bicarbonate dialysate, severe alkalemia in ESRD was successfully corrected with the use of lower-bicarbonate dialysate during hemodialysis in this case. Clinicians should be aware of the option to use lower-bicarbonate dialysate to correct alkalosis in patients with ESRD on hemodialysis.
